# Proximal interphalangeal replantation with arthrodesis facilitates favorable esthetics and functional outcome

**DOI:** 10.1186/s13032-015-0028-z

**Published:** 2015-10-27

**Authors:** Masaki Fujioka, Kenji Hayashida

**Affiliations:** Department of Plastic and Reconstructive Surgery, National Hospital Organization Nagasaki Medical Center, 1001-1 Kubara 2, 856-8562 Ohmura City, Japan

**Keywords:** Finger amputation, Finger avulsion, Hand surgery outcomes, Replantation, Revascularization

## Abstract

**Purpose:**

Management of finger amputations of the proximal interphalangeal (PIP) joint is still controversial. Regrettably, injured PIP joints seldom regain normal active motion; thus, many investigators recommend revision amputation with skeletal injury at or proximal to the PIP joint. We report the functional outcome of patients with replantation or revascularization following complete or incomplete amputations of the PIP joint.

**Methods:**

A total of 15 digital replantations or revascularization were performed on 11 patients (9 males and 2 females, age, 26–69 years) with severe finger injuries at the PIP joint at our Medical Center from 2010 through 2012. Seven patients with 10 complete amputations underwent replantations, and 4 with 5 incomplete avulsion amputations underwent revascularization. PIP arthrodesis was performed in all cases. Routine postoperative evaluation was performed in 13 successfully treated patients.

**Results:**

The 13 successfully treated cases were tracked over a follow-up of 12 to 55 months. Arthrodesis of PIP caused significantly lower total active range of motion (TAM; 85–120°). The mean DASH score was 37/100 (range: 10–64 points). Although mobility is poorer in PIP replantations, adequate PIP joint fixation improves DASH score and hand function.

**Conclusions:**

PIP replantation along with arthrodesis at a functional position for a finger amputation should be performed when the patient wishes to undergo replantation, which facilitates patient satisfaction.

## Findings

Regrettably, injured PIP joints seldom regain normal active motion. A total of 15 digital replantations or revascularization were performed for the severe finger injuries at the PIP joint, and 13 successfully treated cases were evaluated. Although mobility is poorer in PIP replantations, adequate PIP joint fixation improves DASH score and hand function. PIP replantation along with arthrodesis at a functional position for a finger amputation facilitates patient satisfaction.

## Introduction

Eighty to ninety percent survival rate of the replantation of amputated fingers is reported in the literature [1, 2]. However, avulsion injuries are commonly cited as having poor functional outcomes after replantation, especially in the case of complete amputation or when there is damage to the proximal phalanx or the proximal interphalangeal (PIP) joint [3–5]. Thus, many investigators recommend revision amputation with skeletal injury at or proximal to the PIP joint [6]. However, a large number of patients desire the replantation of their amputated fingers, and recent functional outcomes of sensibility and range of motion after replantation of finger avulsion injuries are better than what is historical data [6].

We report the functional outcome of patients with replantation or revascularization following complete or incomplete avulsion amputations at the PIP joint.

## Patients and methods

A total of 15 digital replantations or revascularization were performed on 11 patients (2 women and 9 men, age, 26–69 years) with severe finger injuries at the PIP joint at our Medical Center from 2010 through 2012 (Table 1). These included 6 index fingers, 5 middle fingers, 3 ring fingers and a little finger. The injuries resulting in amputations were as follows: 10 avulsions, 5 crushes and no cleanly severed cases. Seven patients with 10 complete amputations underwent replantations, and 4 with 5 incomplete amputations underwent revascularization. PIP arthrodesis at an angle of 45° was performed in all cases. Among these patients, 2 developed postoperative necrosis. Thirteen patients with successfully replanted fingers returned for testing that consisted of an interview using a patient-centered questionnaire (The Disabilities of the Arm, Shoulder and Hand (DASH) score) and physical examinations of the range of motion.

**Table 1:** Patient who underwent digital replantations or revascularization with severe finger injuries at the PIP joint. PIP arthrodesis at angle of 45° was performed in all cases

	Age Sex	Amputation type	Injury type	Amputated finger	Total Active Motion	Dash score
1	53 M	Complete	Avulsion	index, middle, right, little	Index 60, middle 60, ring 60, little 60	23
2	44 M	Complete	Avulsion	Index	90	11
3	57 F	Incomplete	Crush	Index	80	15
4	69 M	Incomplete	Avulsion	index, middle	Index 80, middle 80	40
5	26 M	Incomplete	Avulsion	Index	152	25
6	65 M	Complete	Avulsion	middle	85	35
7	59 M	Complete	Crush	Index	32	64
8	58 M	Complete	Crush	middle	98	20
9	56 M	Complete	Avulsion	middle	80	22
10	59 F	Incomplete	Crush	ring	112	10
11	59 M	Complete	Crush	ring	85	12

## Results

The mean active flexion of the PIP joint was 0° (arthrodesis), mean total active arc of motion (TAM) was 92 (range: 32–152) degrees and the mean DASH score was 37/100 (range: 10–64 points).

## Case reports

### Case 1

**Fig. 1:**
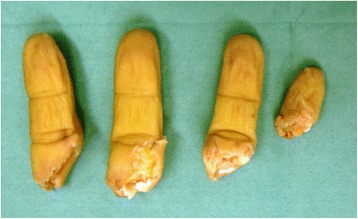
Case 1. A 53-years-old man sustained complete amputation to the left index, middle, ring and little fingers. View od amputated fingers

**Fig. 2:**
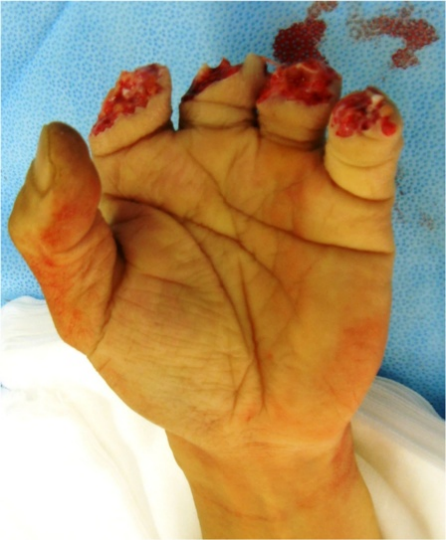
Case 1. View of injured hand without four finders

**Fig. 3:**
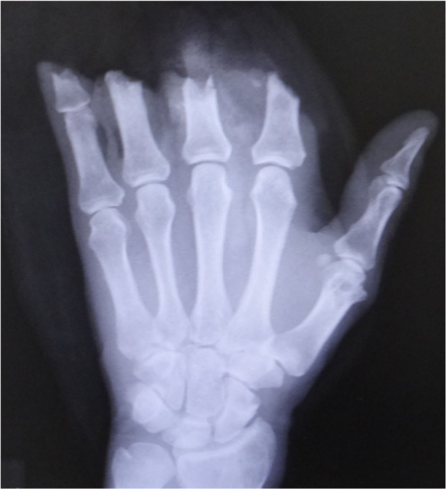
Case 1. An X-ray image of injured hand

**Fig. 4:**
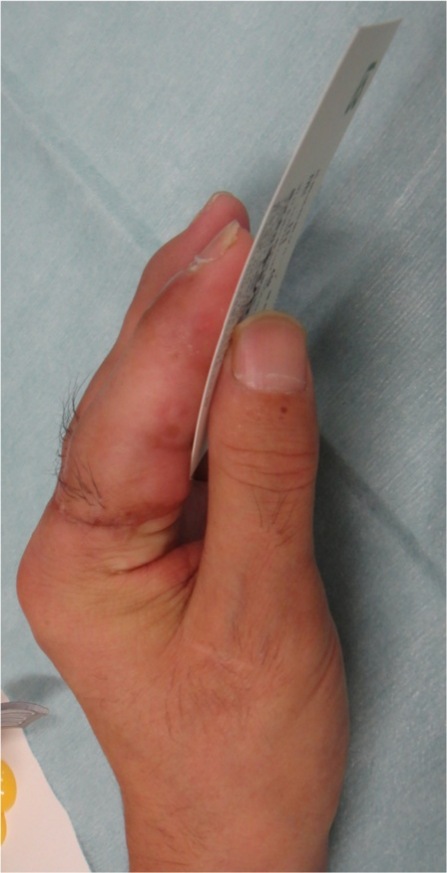
View of injured hand 3 months after the replantation surgery. The patient could not pinch because the PIP joints were fixed straight

**Fig. 5:**
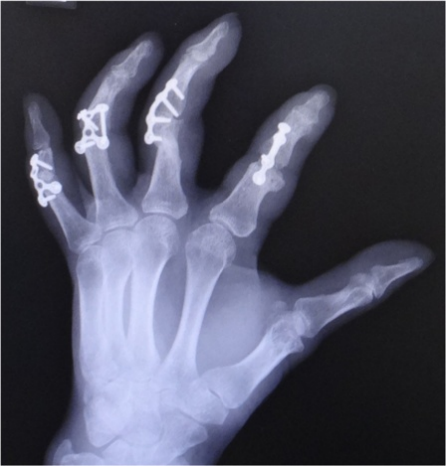
An X-ray image after arthrodesis with micro-plates at an angle of 45° of the PIP joints

**Fig. 6:**
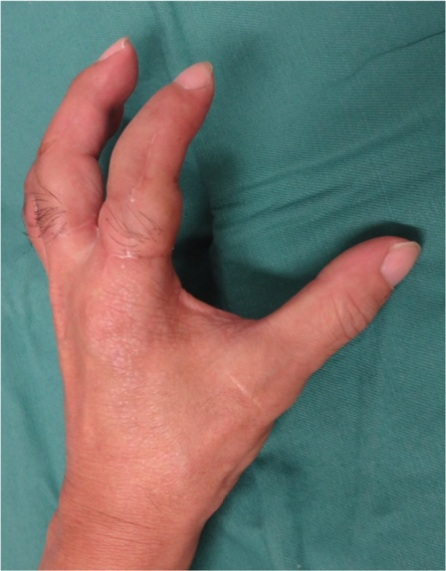
View of injured hand 3 months after the secondary surgery. The PIP joints were fixed at an angle of 45 degrees

**Fig. 7:**
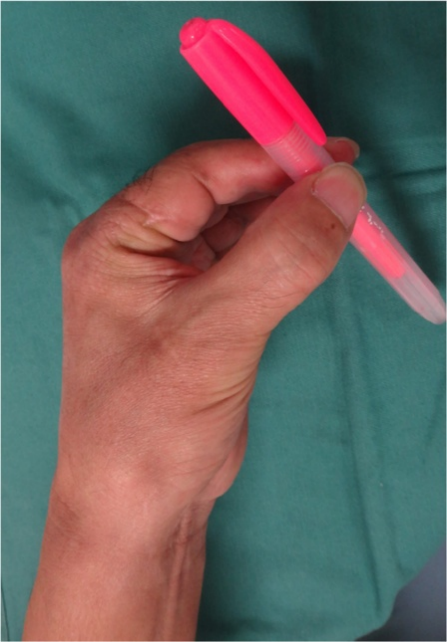
View of injured hand 3 months after the secondary surgery. The patient acquired pinch function

A 53-year-old man sustained an avulsion amputation to the left index, middle, ring and little fingers due to being caught in the cutter of the aluminum sash (Figs. 1, 2, 3). The amputated levels of the index, middle and ring fingers were at the proximal phalangeal bone and fracture of PIP joints were recognized. That of the little finger was at the distal interphalangeal joint. Replantation surgery of all amputated fingers was performed in compliance with the patient’s wishes. Each arthrodesis was performed with two K-wires. All replanted fingers survived, however, the patient could not pinch because the PIP joints were fixed straight (Fig. 4). The active TAM was 60° and the DASH score was 79 points. The patient underwent tenolysis and re-arthrodesis of PIP joints to a functional position, which consisted of PIP joints 45°,12 months later. This position resulted in the patient having satisfactory postoperative pinch function and appearance, while the DASH score improved to 23 points (Figs. 5, 6, 7).

### Case 2

**Fig. 8:**
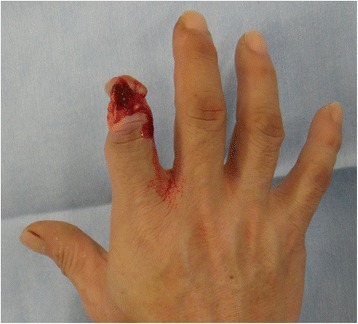
Case 2. A 44-year-old man suffered from complete amputation at the level of the middle phalangeal bone of the right index finger along with crush fracture on the PIP joint

**Fig. 9:**
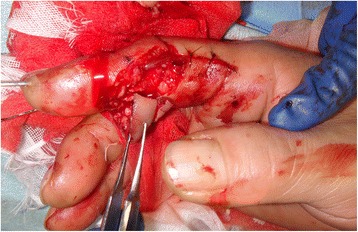
Intra-operative photograph shows vascular anastomosis and PIP fixation with K-wire

**Fig. 10:**
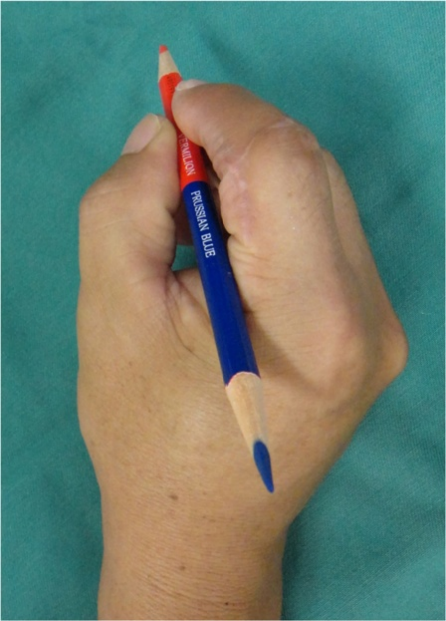
The picture shows good form and acceptable function in opposition 13 months after replantation

A 44-year-old man suffered from complete amputations at the level of the middle phalangeal bone of the right index finger along with the crush fracture on the PIP due to being caught in the food cutter (Fig. 8). The patient underwent replantation and arthrodesis at an angle of 45° of the PIP joint (Fig. 9). Good form and acceptable function in opposition and grasp were evident 13 months after the procedure, while the active TAM was 90° and the DASH score was 11 points (Fig. 10).

### Case 3

**Fig. 11:**
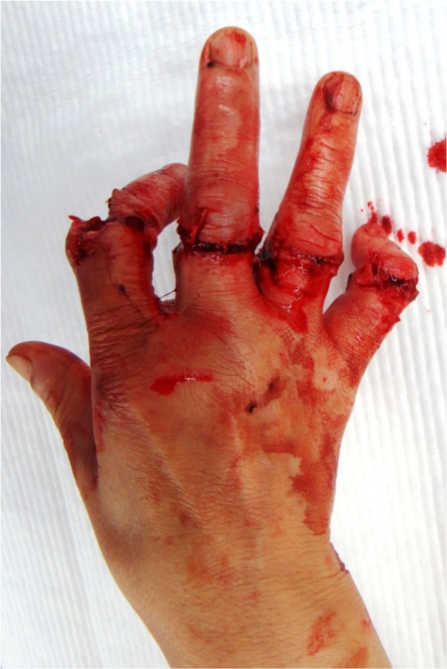
Case 3: A 57-year-old woman suffered from incomplete amputation at the level of the PIP joint of the right index finger without distal circulation, along with the crush fracture on the proximal phalangeal bone of middle, ring and little fingers

**Fig. 12:**
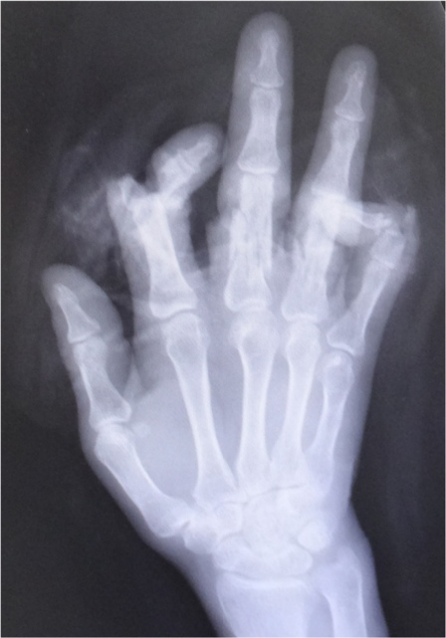
Case 3. An X-ray image of unjured hand

**Fig. 13:**
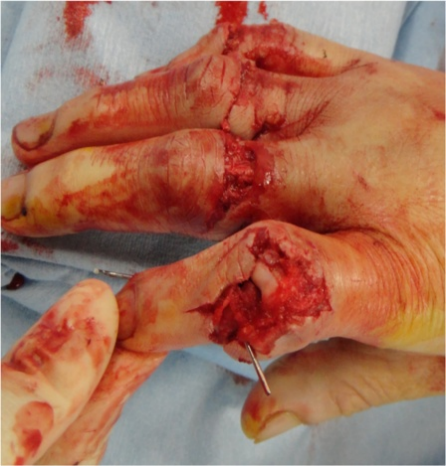
Intra-operative photograph shows exposure of PIP joint

**Fig. 14:**
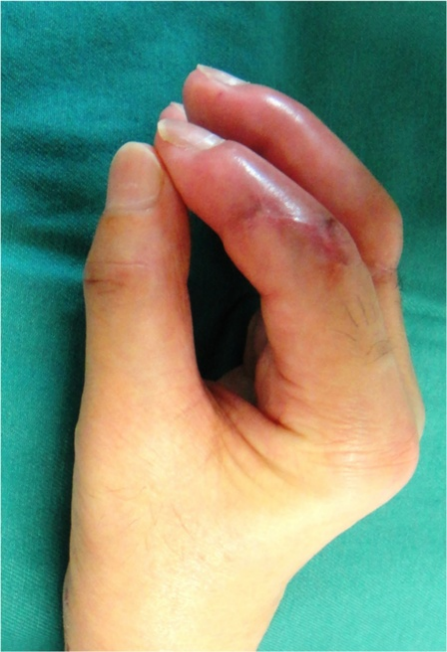
The picture 15 months after surgery shows good form and acceptable function in opposition

**Fig. 15:**
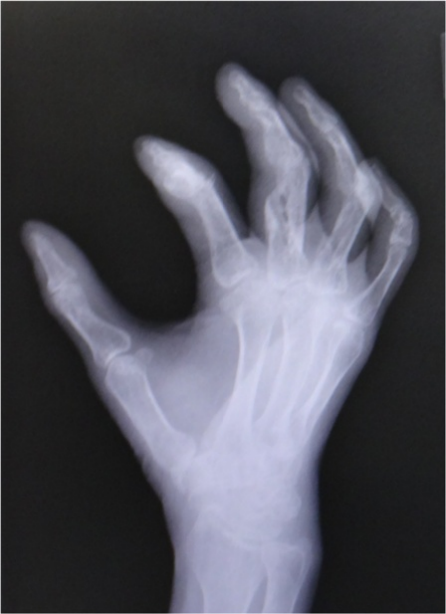
An X-ray image 15 months after surgery shows bone union of PIP joint of index finger

A 57-year-old woman suffered from incomplete amputations at the level of the PIP joint of the right index finger without distal circulation, along with crush fracture on the proximal phalangeal bone of middle, ring and little fingers due to being caught in the rotor of a food processor (Fig. 11, 12). The patient underwent revascularization and PIP joint arthrodesis at an angle of 45° of the index finger, and reduction and K-wire fixation of the other three fractured fingers (Fig. 13). Good form and acceptable function in opposition and grasp were evident 15 months later, while the TAM was 80° and the DASH score was 15 points (Fig. 14, 15).

## Discussion

The salvage of amputated fingers has become a common procedure owing to advances in microsurgical technique. When the amputation occurred at the level of the DIP joint, replantation showed advantages including a one-stage procedure, adequate sensibility without painful neuroma, good metacarpophalangeal and PIP joint motion, and a cosmetically pleasing outcome compared with conventional stamp plasty [6]. However, it is still difficult to achieve satisfactory functional results in cases of replantation or revascularization at the level of the PIP joint. Avulsion amputations at the PIP joint are likely to result in permanent disability of the hand [7]. Thus, many investigators considered that replantation of a single finger amputated proximal to the flexor digitorum superficialis insertion was seldom indicated [1]. Soucacos et al. evaluated the functional outcome of 67 successfully replanted single-digit amputations, and concluded that the indications for replantation of a single-digit amputation should be as follows: 1) amputation distal to the insertion of the flexor digitorum superficialis; 2) ring injuries type II and IIIa; and 3) amputations at the level of or distal to the DIP joint [8]. Davis and Chung recommended revision amputation with skeletal injury at or proximal to the PIP joint in patients who are unwilling to accept a poor functional result [6].

However, when a patient with finger amputation proximal to the PIP joint wants to undergo replantation surgery, what should we do? In our experience, most patients who visited our emergency unit with amputated fingers desired replantation, even though they were informed about the unsatisfactory postoperative hand function. Our study showed that, although mobility is poorer in PIP replantations, patients can acquire a pinch function when the finger can be fixed in a functional position. Adequate PIP joint fixation improves DASH score and hand function. Hattori et al. studied 46 distal amputations in which 20 out of 23 patients in the replantation group always used the replanted finger for activities of daily living, whereas only 9 out of 23 patients in the amputation closure group always used the affected fingers. The replantation group also had less pain and a better DASH score. They concluded that replantation not only facilitated more favorable esthetics, but also a better functional outcome [9].

Of course, most hand surgeons would expect preservation of the PIP joint. However, as we showed in Case 1, a destroyed joint seldom regains normal activity, which would cause a significant disadvantage for the functional recovery of a hand. Thus, we recommend primary arthrodesis at a functional position following replantation, which would provide the best functional result along with shortening the treatment period.

## Conclusion

We believe that PIP replantation along with arthrodesis at a functional position should be performed when the patient wishes to undergo replantation. This method provides both favorable esthetics and an acceptable functional outcome, facilitating patient satisfaction.

### Ethical considerations

The authors herewith certifies that they are responsible for the contents of the manuscript. They have complied with the guidelines for conducting research in human subjects. The procedures are in accordance with the ethical standards of institutional committee on human experimentation of National Hospital Organization Nagasaki Medical Center.

The procedures followed were in accordance with the ethical standards of the National Institutes of Health, our institutional committee on human experimentation, and with the Helsinki Declaration of 1975, as revised in 1983.

## Abbreviations

PIP: Proximal interphalangeal
